# High‐Intensity Alternating Current Stimulation as an Add‐On to Multidisciplinary Intensive Rehabilitation for Parkinson's Disease: A Randomized Controlled Trial

**DOI:** 10.1002/cns.70909

**Published:** 2026-04-27

**Authors:** Ting‐ting Hou, Hong‐yu Zhang, Ke‐ke Chen, Zhao‐hui Jin, Tian Zhang, Lin Qi, Cui‐ping Xue, Qiao‐xia Zhen, Zhen‐zhen Li, Hong‐jiao Yan, Yi Zhen, Xia An, Jia Du, Yuan Su, Cui Liu, Qi‐ping Wen, Xiao‐yan Yan, Bastiaan R. Bloem, Bo‐yan Fang

**Affiliations:** ^1^ Beijing Rehabilitation Medicine Academy Capital Medical University Beijing China; ^2^ Parkinson Medical Center, Beijing Rehabilitation Hospital Capital Medical University Beijing China; ^3^ Department of Radiology, Beijing Rehabilitation Hospital Capital Medical University Beijing China; ^4^ Peking University Clinical Research Institute Peking University First Hospital Beijing China; ^5^ Department of Neurology, Center of Expertise for Parkinson & Movement Disorders Radboud University Medical Centre; Donders Institute for Brain, Cognition and Behaviour Nijmegen HB the Netherlands

**Keywords:** multidisciplinary intensive rehabilitation therapy, parkinson's disease, quality of life, transcranial alternating current stimulation

## Abstract

**Aims:**

In this randomized, double‐blind, sham‐controlled trial, we explored whether high‐intensity transcranial alternating current stimulation (Hi‐tACS), as an add‐on to multidisciplinary intensive rehabilitation therapy (MIRT), could yield more durable quality of life (QoL) improvements than MIRT alone.

**Methods:**

Sixty patients with Parkinson's disease (PwP) (Hoehn and Yahr stages 1–3, aged 45–70) were assigned (1:1) to receive 10 days of MIRT with either twice‐daily Hi‐tACS (15 mA, 77.5 Hz, 40 min/session) or sham stimulation. The primary outcome was the longitudinal change in the 39‐item Parkinson's Disease Questionnaire (PDQ‐39) total score from baseline to 4, 12, and 24 weeks after intervention. Secondary outcomes included changes in motor and non‐motor symptom scales. Adverse events were monitored throughout the intervention period.

**Results:**

Generalized estimating equation analysis revealed a significant group‐by‐time interaction for PDQ‐39 total scores (*P*_*group × time*_ = 0.008). The Hi‐tACS+MIRT group demonstrated significantly greater reductions in PDQ‐39 scores than the sham‐Hi‐tACS+MIRT group at 4 weeks (T2: effect size = 0.68, 95% CI: 0.14–1.21; *P*_*FDR*_ = 0.009), 12 weeks (T3: effect size = 1.43, 95% CI: 0.80–2.05; *P*_*FDR*_ = 0.003), and 24 weeks (T4: effect size = 1.26, 95% CI: 0.65–1.85; *P*_*FDR*_ < 0.001). In addition, the Hi‐tACS+MIRT group exhibited superior improvements in depression and apathy. Adverse events were mild and infrequent, with no serious adverse events reported.

**Conclusions:**

This trial demonstrated that the Hi‐tACS add‐on to MIRT could maintain long‐term improvements in QoL and improve non‐motor symptoms for PwP, providing a new strategy for PwP rehabilitation.

## Graphical abstract

This trial demonstrates that high‐intensity transcranial alternating current stimulation (Hi‐tACS) add ‐ on to multidisciplinary intensive rehabilitation training (MIRT) can lead to greater improvement in quality of life in Parkinson's disease for 24 weeks.

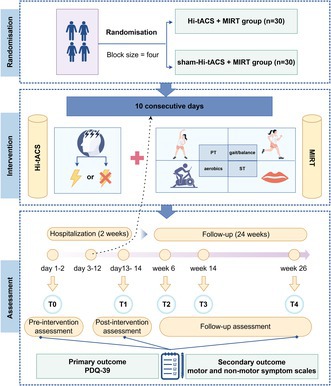

## 1 Introduction

Parkinson's disease (PD) progressively diminishes the quality of life (QoL) through the manifestation of both motor and non‐motor symptoms [1]. While conventional rehabilitation can improve the QoL for patients with PD (PwP), these approaches often necessitate prolonged intervention periods and typically result in only transient improvements [2, 3]. Multidisciplinary intensive rehabilitation therapy (MIRT) overcomes the challenge of prolonged intervention by providing high‐intensity, multimodal interventions over a shorter period. Our previous study demonstrated significant motor improvements following a two‐week MIRT protocol [4]. However, these benefits lasted only 3 months post‐treatment [4, 5]. Based on the “central–peripheral–central” closed‐loop rehabilitation framework [6], we propose that combining neuroplasticity‐promoting interventions with rehabilitation can lead to more sustained improvements in the symptoms of PwP.

Transcranial alternating current stimulation (tACS) is a promising technique for modulating neuroplasticity. By delivering rhythmic, sinusoidal current, tACS can entrain endogenous neural oscillations and promote synaptic adaptation [7]. Previous studies on low‐intensity tACS have not shown long‐term efficacy for PD, possibly due to inadequate penetration and an inability to effectively modulate subcortical neural networks [8]. As a potential means to address these limitations, high‐intensity tACS (Hi‐tACS: 77.5 Hz, 15 mA), as utilized by the Nexalin device, is capable of delivering current to deeper brain structures and enhancing neurochemical signaling, including the release of beta‐endorphin [9]. To date, only one randomized clinical trial has evaluated the efficacy of Hi‐tACS in PwP. While it showed no significant improvement in motor and psychological symptoms compared with the sham stimulation group after 10 sessions of treatment alone, a trend towards improvement and good safety was observed [10].

Therefore, we conducted a randomized, double‐blind, sham‐controlled trial to investigate whether a two‐week, more frequent Hi‐tACS protocol combined with MIRT could yield more durable improvement in QoL for PwP.

## 2 Methods

### 2.1 Design

The study was conducted at a single centre, Beijing Rehabilitation Hospital, Capital Medical University (Beijing, China) with the hospital's Department of Scientific Research Management overseeing trial implementation and compliance. All participants provided written informed consent, and data anonymity was ensured throughout the study. The protocol was approved by the Ethics Committee of Beijing Rehabilitation Hospital, Capital Medical University (Ethical review No. 2023kky‐064) and reported in accordance with CONSORT guidelines. The trial was registered with ChiCTR (ChiCTR2300071969). Details of the trial design are outlined elsewhere (Supinfo S1) [11].

### 2.2 Participants

From October 2023 to January 2025, patients aged 45–70 years with PD were recruited (PD was diagnosed according to the 2015 Movement Disorder Society criteria; disease severity Hoehn and Yahr stages 1–3). Inclusion criteria included clinically relevant motor impairment or functional limitations indicating a rehabilitation need, stable medication use for over 2 weeks, no history of deep brain stimulation, independent ambulation and self‐care, primary school education or above, and provision of written informed consent. Exclusion criteria comprised atypical or secondary Parkinsonism (e.g., vascular, drug‐induced), other neurological conditions affecting cognition or motor function, epilepsy, contraindications to tACS, MRI incompatibility (e.g., claustrophobia, metallic implants), major organ dysfunction, participation in other clinical trials, implantation in vivo, or inability to comply. Eligible participants who provided consent were randomized and received treatment. Participants were withdrawn if they revoked consent or discontinued treatment.

### 2.3 Randomization and Masking

Eligible participants were randomly assigned using a block randomization method (block size = 4) via SPSS 27.0 (IBM) by an independent statistician. Participants were assigned in a 1:1 ratio to either the experimental group (Hi‐tACS+MIRT) or the control group (sham‐Hi‐tACS+MIRT) for a two‐week inpatient intervention. Allocation concealment was ensured through automated electronic coding managed by a blinded member of the Data Monitoring Committee (DMC). All randomization records, including encrypted device codes (A/B), were securely stored in opaque sealed envelopes opened only during stimulation sessions.

Identical Nexalin devices delivered either active (77.5 Hz, 15 mA) or sham (no current) stimulation, labeled as “Device A” or “Device B”, respectively, to maintain blinding. Operators and investigators were blinded to group allocation, with password‐protected access restricted to the DMC. Sham stimulation mimicked the active protocol (including auditory/visual cues and ramp phases), without current delivery. Blinding was assessed immediately following the intervention in both groups.

### 2.4 Intervention

#### 2.4.1 High‐Intensity Transcranial Alternating Current Stimulation

Participants received twice‐daily 40‐min Hi‐tACS/sham sessions (with a six‐hour interval) over ten consecutive days (20 total sessions). FDA‐cleared Nexalin devices [12] delivered 77.5 Hz square‐wave stimulation at 15 mA (zero‐to‐peak) via frontal (Fpz/Fp1/Fp2, 4.45 × 9.53 cm) and bilateral mastoid electrodes (3.18 × 3.81 cm). Sessions included 180‐s ramp‐up and 12‐s ramp‐down phases. Sham devices replicated active protocols without current delivery. Electrodes were positioned by a trained staff member.

#### 2.4.2 Multidisciplinary Intensive Rehabilitation Therapy

In conjunction with Hi‐tACS intervention, all participants completed a ten‐day MIRT rehabilitation program comprising four daily modules: (1) physical therapy incorporating warm‐up routines, active/passive stretching, and flexibility training in 40‐min group sessions (four participants/group); (2) computerized gait/balance rehabilitation using C‐MiLL (Motek, Netherlands) and Balance Tutor (Meditouch, Israel) systems with 30‐min individualized sessions; (3) aerobic conditioning via NuStep T5XR cross‐trainer (30‐min sessions); and (4) speech therapy delivered by certified therapists in 60‐min group sessions. Each therapeutic module was conducted by the same certified therapists to ensure consistency in therapeutic outcomes.

### 2.5 Outcomes

Each participant completed a 26‐week study comprising a baseline assessment (T0) 2 days before hospitalization, a 10‐day intervention, and follow‐up evaluations at the end of the intervention (T1; days 13–14) and at 4 (T2), 12 (T3), and 24 (T4) weeks post‐intervention.

To minimize the potential influence of dopaminergic medication, all clinical assessments were conducted during the medication‐off state (≥ 12 h after levodopa withdrawal). Only the “off” condition was used for all analyses and outcome evaluations. Follow‐up assessments were conducted either via an online system or during outpatient visits.

### 2.6 Primary Outcome

The primary outcome was the 39‐item Parkinson's Disease Questionnaire (PDQ‐39), which assessed changes in QoL from T0 to T2, T3, and T4. This is a validated self‐report tool covering eight domains (mobility, activities of daily living, emotional well‐being, stigma, social support, cognition, communication, and bodily discomfort) that evaluate patients' status over the preceding month. Therefore, it was not appropriate to assess the PDQ‐39 immediately after the 10‐day intervention (T1). Accordingly, PDQ‐39 was assessed only at T0, T2, T3, and T4. Each item was rated on a five‐point Likert scale (never = 0, always = 4). Subscale scores were calculated as percentages of the maximum possible score, and the total PDQ‐39 score was the average of the eight subscales. Lower scores indicate better QoL [13].

### 2.7 Secondary Outcomes

#### 2.7.1 Secondary Outcomes Encompassed Both Motor and Non‐Motor Symptom Scales

The motor symptom scales included: (1) Part 3 of the Movement Disorders‐Unified Parkinson's Disease Rating Scale (MDS‐UPDRS III), an 18‐item scale designed to assess motor function in PwP [14]; (2) the Modified Parkinson Activity Scale (M‐PAS), which evaluates key activity limitations in PwP [15]; (3) the Berg Balance Scale (BBS), which assesses an individual's ability to actively control their centre of gravity during daily functional activities, providing a comprehensive evaluation of both dynamic and static balance in sitting and standing positions [16]; and (4) the Timed Up and Go Test (TUG), a tool for the comprehensive assessment of balance ability, walking speed, turning ability, and lower limb muscle strength in the elderly, effectively evaluating their daily activity capabilities [17].

The non‐motor symptom scales included: (1) the Non‐Motor Symptoms Scale (NMSS), a 30‐item clinician‐rated tool that covers nine symptom domains over the past month, scored by multiplying severity (0–3) and frequency (1–4) [18]; (2) the Geriatric Depression Scale (GDS), a 30‐item self‐report measure for assessing depression in older adults [19]; (3) the Hamilton Depression Scale (HAMD), a multi‐item questionnaire utilized to indicate depression levels and guide recovery evaluation [20]; (4) the Hamilton Anxiety Scale (HAMA), comprising 14 items that measure the severity of anxiety symptoms [21]; and (5) the Modified Apathy Estimate Scale (MAES), which assigns higher scores to indicate greater apathy [22].

Assessments of motor and non‐motor outcomes were scheduled at timepoints T0, T1, T2, T3, and T4. However, follow‐up for motor outcomes requiring professional in‐person evaluation was significantly impacted by participants' inability to return for face‐to‐face visits at T2, T3, and T4. Consequently, these specific in‐person motor assessments were analyzed only at T0 and T1. For non‐motor outcomes, data were collected at T0, T2, T3, and T4. Assessment at T1 was excluded from non‐motor analysis because the standardized scales used primarily reflect the patient's status over the preceding month, making assessment immediately post‐intervention (T1) methodologically inappropriate.

### 2.8 Adverse Events

Adverse events were monitored during the course of treatment and through interviews to identify conditions such as discomfort, pain, or injury. Participants were instructed to promptly report any study‐related adverse events to the research team.

### 2.9 Sample Size

The sample size was calculated using PASS 11 software. Based on previous literature reporting PDQ‐39 outcomes in PwP [23, 24], we estimated a standard deviation of 10.0 and an expected between‐group difference of 9.0 points (Cohen's *d* ≈0.9), a value chosen to reflect a robust clinical benefit corresponding to approximately twice the established minimal clinically important difference (MCID) [25]. With a statistical power of 0.9 and a significance level (α) of 0.05, 27 participants per group were required. Accounting for a 10% attrition rate, each group was planned to include 30 patients, totaling 60 participants.

### 2.10 Data Analysis

Statistical analyses were performed using SPSS 27.0 (IBM). Data normality was assessed with the Shapiro–Wilk test. Continuous variables are presented as mean ± standard deviation or median (interquartile range), and categorical variables as counts (percentages). All tests were two‐tailed, with significance set at *p* < 0.050. Analyses adhered to the intention‐to‐treat (ITT) principle, with multiple imputation applied for missing values. Missing data for the primary outcome (PDQ‐39) was low, with a total of 4 participants (6.7%) dropping out during the trial. The missing data mechanism was assumed to be missing at random (MAR), and multiple imputation was performed using SPSS. Five imputation cycles were conducted. For the final analysis, the results from all imputed datasets were pooled according to Rubin's rules.

For motor outcomes at T0 and T1, between‐group differences were analyzed using independent samples *t*‐tests or Mann–Whitney U tests, as appropriate, while within‐group differences were examined using paired samples *t*‐tests or Wilcoxon signed‐rank tests.

Longitudinal changes in PDQ‐39 scores and non‐motor outcomes were analyzed using generalized estimating equation (GEE) models with a first‐order autoregressive (AR (1)) working correlation structure. The dependent variable was the outcome score, and the independent variables included group, time, and the group‐by‐time interaction. Baseline measurements (T0) were included as the first repeated observation in the model. The effect sizes reported represent model‐based standardized effect estimates derived from the GEE models for longitudinal data and independent tests for single time points, rather than simple raw score differences. The direction of these effect estimates was calculated as the sham‐Hi‐tACS+MIRT group minus the Hi‐tACS+MIRT group; therefore, positive values indicate higher scores in the sham‐Hi‐tACS+MIRT group relative to the Hi‐tACS+MIRT group.

### 2.11 Role of the Funding Source

The funder of the study had no role in study design, data collection, data analysis, data interpretation, or writing the report.

## 3 Results

### 3.1 Participants

**FIGURE 1:**
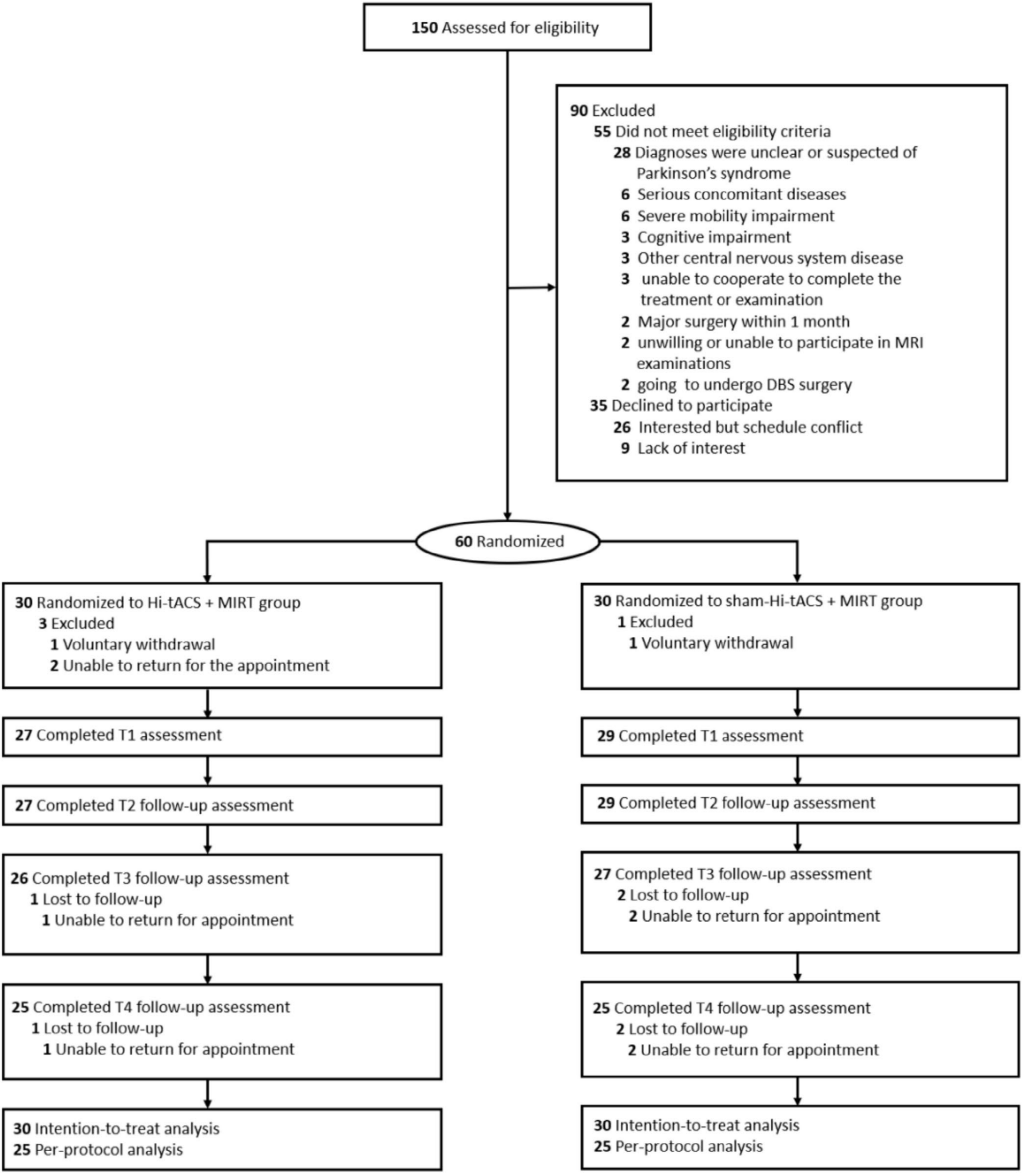
CONSORT diagram of study flow.

Sixty eligible participants were enrolled and assigned randomly to the two groups (1:1 ratio). During treatment, three participants in the Hi‐tACS+MIRT group dropped out. Among them, one withdrew voluntarily, and two were unable to attend appointments on time. One participant in the sham‐Hi‐tACS+MIRT group dropped out voluntarily. During follow‐up, six participants (two from Hi‐tACS+MIRT and four from sham‐Hi‐tACS+MIRT) withdrew due to scheduling conflicts. Consequently, 50 participants (25 per group) completed the entire trial (Figure 1). Baseline demographics were well‐balanced between groups (Table 1), and baseline demographics in the original sample were consistent with the ITT population (Table S1).

**TABLE 1:** Baseline demographics in the ITT sample.

Characteristics	All (*n* = 60)	Hi‐tACS+MIRT (*n* = 30)	sham‐Hi‐tACS+MIRT (*n* = 30)
Age, median (IQR), y	64 (57, 68)	65.5 (60, 69)	61.5 (56, 68)
Sex, *n* (%)			
Male	26 (43.3)	13 (43.3)	13 (43.3)
Female	34 (56.7)	17 (56.7)	17 (56.7)
Affected side, *n* (%)			
Left	24 (40)	15 (50)	9 (30)
Right	36 (60)	15 (50)	21 (70)
Hoehn & Yahr (on^a^), *n* (%)			
1.0	2 (3.3)	2 (6.7)	0 (0)
1.5	14 (23.3)	8 (26.7)	6 (20)
2.0	32 (53.3)	17 (56.7)	15 (50)
2.5	5 (8.3)	1 (3.3)	4 (13.3)
3.0	7 (11.7)	2 (6.7)	5 (16.7)
Hoehn & Yahr (off^b^), *n* (%)			
1.0	0 (0)	0 (0)	0 (0)
1.5	11 (18.3)	7 (23.3)	4 (13.3)
2.0	31 (51.7)	17 (56.7)	14 (46.7)
2.5	8 (13.3)	4 (13.3)	4 (13.3)
3.0	10 (16.7)	2 (6.7)	8 (26.7)
Disease Duration, y	7 (5,8)	7 (5, 8)	6 (5.75, 9)
Treatment duration, y	5.6 ± 2.6	5.6 ± 2.3	5.6 ± 2.8
Years of education, median (IQR), y	12.5 (12, 15.8)	13 (11, 16)	12 (12, 15.25)
LEDD	546.56 ± 212.11	539.93 ± 227.64	553.18 ± 199.05

Abbreviations: IQR, interquartile range; LEDD, Levodopa equivalent daily dose.

^a^ “On” represents “on state” of levodopa treatment (1–2 h post‐levodopa).

^b^ “Off” represents “off state” of levodopa treatment (≥ 12 h post‐levodopa).

### 3.2 Primary Outcome

For the PDQ‐39 total score, GEE analysis revealed a significant group‐by‐time interaction (*P*_*group × time*_ = 0.008), indicating different longitudinal trajectories between groups.

**FIGURE 2:**
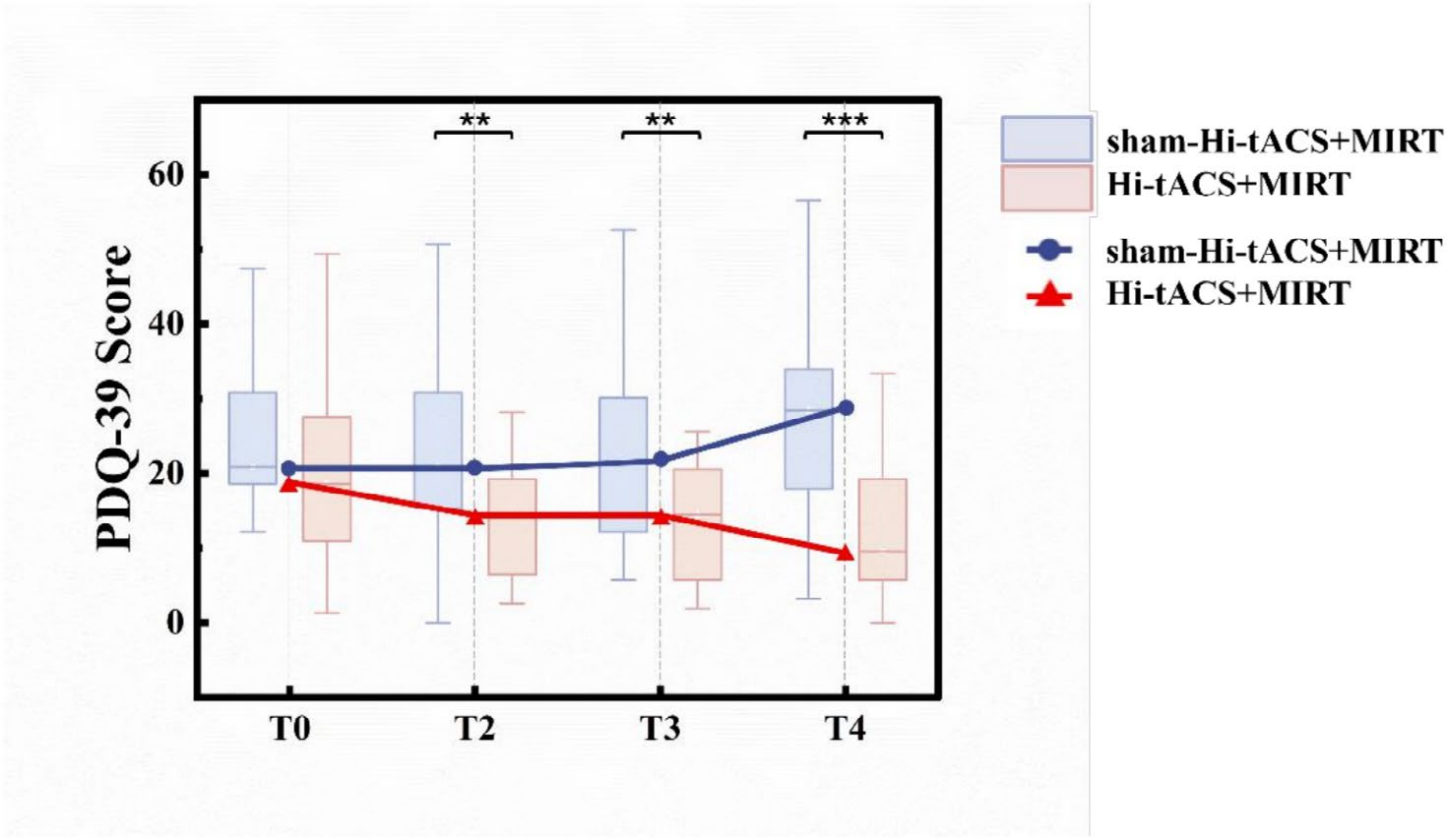
Differences between the two groups in primary outcomes (PDQ‐39 scores). Between‐group differences at each time point (FDR‐corrected) are shown following a significant time and group interaction effect. *p < 0.05; **p < 0.01; ***p < 0.001.

The Hi‐tACS+MIRT group demonstrated significantly greater reductions in PDQ‐39 scores than the sham‐Hi‐tACS+MIRT group at T2 (effect size = 0.68, 95% CI: 0.14–1.21; *P*_*FDR*_ = 0.009), T3 (effect size = 1.43, 95% CI: 0.80–2.05; *P*_*FDR*_ = 0.003), and T4 (effect size = 1.26, 95% CI: 0.65–1.85; *P*_*FDR*_ < 0.001) (Figure 2). The improvement trends at each time point in the original sample were largely consistent with the ITT sample (Tables S2 and S3). Notably, at the 24‐week follow‐up (T4), the observed median between‐group difference was 18.77 points, which substantially exceeded the expected difference of 9.0 points used for the sample size estimation, confirming the adequacy of the statistical power.

### 3.3 Secondary Outcomes

**FIGURE 3:**
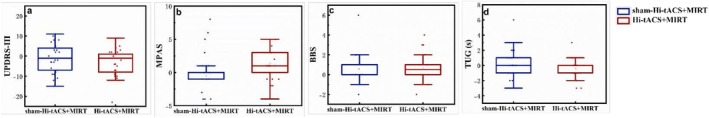
Differences between the two groups in secondary outcomes—motor asessments. The box plots represent the difference scores calculated as post‐intervention (T1) minus Baseline (T0) for: (a) Group differences in the third part of the Movement Disorders‐Unified Parkinson's Disease Rating Scale MDS‐UPDRS III. (b) Group differences in Modified Parkinson Activity Scale (MPAS). (c) Group differences in the Berg Balance Scale (BBS). (d) Group differences in the Timed Up and Go Test (TUG).

None of the motor symptom scales at T0 and T1 demonstrated statistically significant between‐group differences (Figure 3).

**FIGURE 4:**
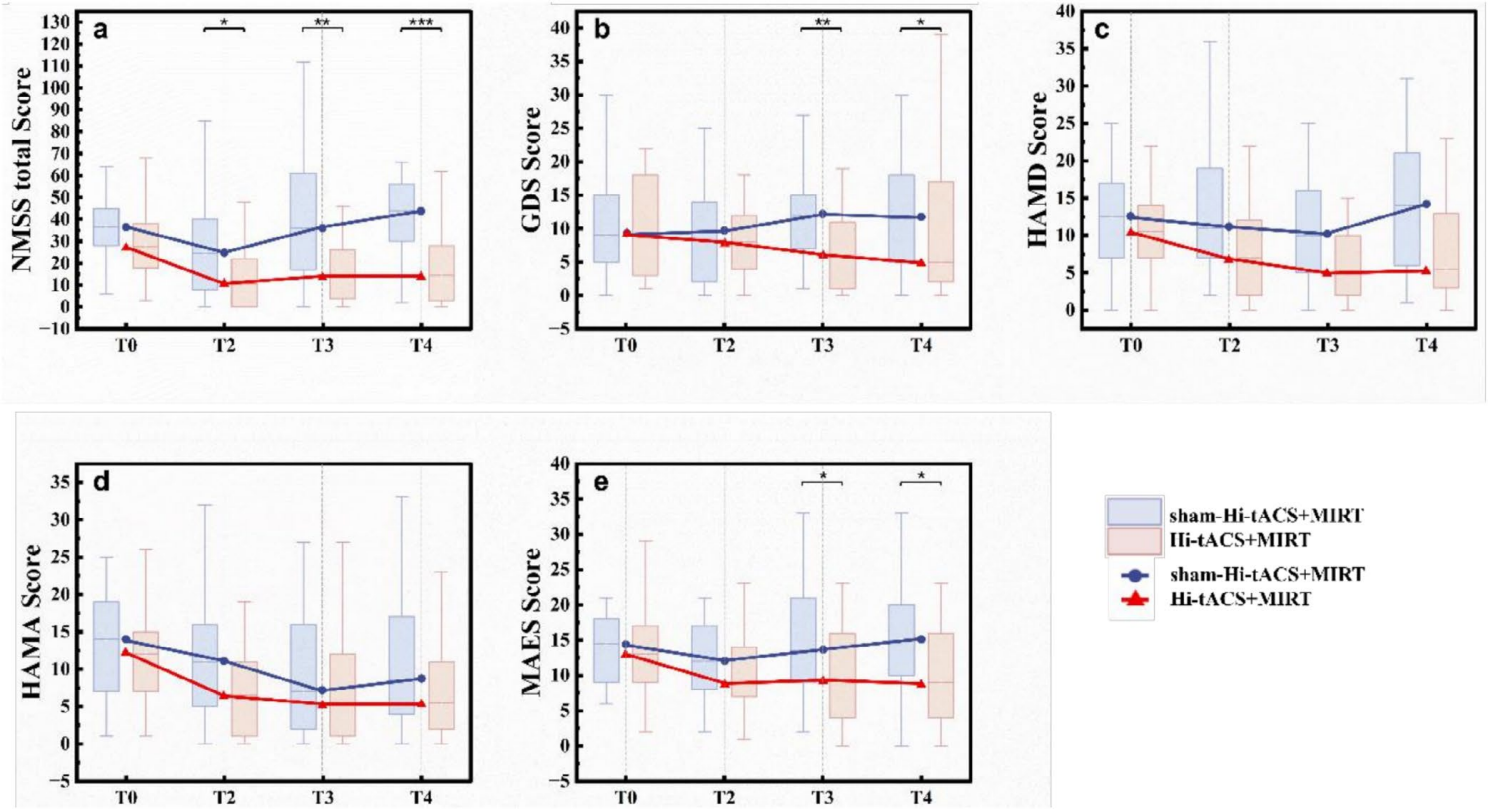
Differences between the two groups in secondary outcomes—non‐motor assessment. (a) Group differences in the Non‐Motor Symptoms Scale (NMSS) scores. (b) Group differences in the Geriatric Depression Scale (GDS) scores. (c) Group differences in Hamilton Depression Scale (HAMD) scores. (d) Group differences in Hamilton Anxiety Scale (HAMA) scores. (e) Group differences in Modified Apathy Estimate Scale (MAES) scores. Between‐group differences at each time point (FDR‐corrected) are shown following a significant time and group interaction effect. *p < 0.05; **p < 0.01; ***p < 0.001.

For non‐motor symptoms, GEE analysis revealed significant group‐by‐time interaction effects in GDS scores (*P*_*group × time*_ = 0.004), MAES scores (*P*_*group × time*_ = 0.028), and NMSS total score (*P*_*group × time*_ = 0.008). Post hoc analyses with FDR correction confirmed that the Hi‐tACS+MIRT group experienced significantly greater improvements than the sham‐Hi‐tACS+MIRT group in GDS and MAES at T3 and T4, and in NMSS total score across all follow‐up time points. No significant interaction was observed for HAMA and HAMD scores (Figure 4). The improvement trends in the original sample were generally consistent with the ITT sample (Tables S4 and S5). Notably, the GDS results demonstrated the most robust consistency, with significant time and group interaction observed in both the ITT and original data analyses.

### 3.4 Adverse Events

During treatment, one participant in the Hi‐tACS+MIRT group (3.7%) and one participant in the sham‐Hi‐tACS+MIRT group (3.4%) reported mild itching at the patch site immediately after intervention, which resolved after cleansing the skin. No serious adverse events were reported (Table S6).

### 3.5 Blinding Efficacy

After Hi‐tACS, 7 participants (26%) believed they had received active stimulation, and 18 participants (67%) claimed they were uncertain. After sham stimulation, 8 participants (28%) thought they had received active stimulation, and 19 participants (66%) stated they were uncertain. The chi‐square test indicated no significant difference in the accuracy of participants' guesses after the two stimulations (*p* = 0.89), suggesting a satisfactory blinding efficacy.

## 4 Discussion

The trial provides novel clinical evidence of the efficacy of Hi‐tACS as an add‐on to MIRT in PD. Regarding the primary outcome, our findings indicate that the Hi‐tACS add‐on to MIRT leads to greater improvements in QoL over 24 weeks in PwP. For the secondary non‐motor outcomes, the Hi‐tACS add‐on to MIRT demonstrated significant benefits, particularly in the domains of depression and apathy, with effects persisting for 24 weeks.

Based on the above findings, we speculate that the observed long‐term improvement of QoL in this study may be closely associated with these non‐motor improvements. While a direct causal link cannot be established, this inference is supported by our previous research, which demonstrated a significant association of PDQ‐39 scores with both the GDS and MAES [24]. Additionally, a previous study also showed that depression was the most significant independent risk factor affecting QoL in PwP [26]. In this study, the GDS results demonstrated robust consistency between the ITT and original sample analyses, supporting the potential antidepressant effects of Hi‐tACS. This finding aligns with previous studies on Hi‐tACS. For example, in a trial involving patients with first‐episode, drug‐naïve major depressive disorder (MDD), Hi‐tACS as monotherapy resulted in a significantly higher remission rate at week 8 compared to sham stimulation [12]. Another recent study further confirmed the efficacy of Hi‐tACS as an add‐on to escitalopram, reporting significantly greater reductions in HAMD‐17 scores and higher response rates in patients with MDD after 4 weeks of treatment [27]. Notably, the improvement in depression observed in PwP in our study was reflected in the self‐reported GDS rather than the clinician‐rated HAMD. This discrepancy suggests that the intervention may exert a preferential effect on the subjective emotional well‐being of PwP. Furthermore, our study not only demonstrates the antidepressant efficacy of combined Hi‐tACS and rehabilitation in PwP, providing clinical evidence supporting its application across different disease contexts, but crucially prolongs the follow‐up period to 24 weeks. Sustained mood improvements and PDQ‐39 score improvements exceeding the established MCID were observed at 24 weeks post‐treatment [25]. This extended observation period is critical for a progressive disorder like PD and provides evidence for the potential of this intervention to confer sustained clinical benefits.

The sustained benefits observed might theoretically be attributed to a combinative interaction between Hi‐tACS and MIRT within a “central–peripheral–central” closed‐loop rehabilitation framework [6]. Based on this theoretical model, we hypothesize that Hi‐tACS may act centrally by delivering high‐intensity and high‐frequency oscillatory currents that enhance neuroplasticity, modulate neuronal excitability, and improve oscillatory coherence across key brain networks, thereby priming restorative structures and compensatory networks for adaptation [28, 29, 30, 31]. This putative centrally primed state is then translated into functional improvements through the repetitive, goal‐oriented peripheral engagement of MIRT, which provides structured sensorimotor input [29]. The resultant refined peripheral performance, in turn, generates enhanced sensory and proprioceptive feedback that further consolidates central neural plasticity, forming a positive reinforcement loop. We further speculate that the stabilization of neurovascular coupling might help secure the metabolic support required for this newly established, efficient brain network [29], underpinning the prolonged improvements in non‐motor symptoms and QoL.

In contrast to the marked non‐motor improvements, the combined intervention did not demonstrate additional motor benefits relative to the sham‐Hi‐tACS+MIRT group. This may be because MIRT itself already exhibits robust efficacy for motor function in PwP, thereby masking any additional measurable improvement from Hi‐tACS [4, 32]. However, although no significant differences were observed between the groups, the Hi‐tACS+MIRT group still demonstrated a better trend towards improvement. Due to unavoidable clinical constraints, long‐term follow‐up assessments for motor function were not feasible in this study. Therefore, the potential motor benefits of Hi‐tACS as an add‐on to MIRT warrant further investigation in larger‐scale studies with extended follow‐up.

Certain limitations to this study must be acknowledged. First, this was a single‐centre trial with a consistent protocol implementation by a dedicated research team. The generalisability of the findings remains to be demonstrated in further multicentre studies. Second, the sample size was modest. While the findings for the primary outcome were robust, the study was underpowered for the original sample analysis due to participant attrition. Specifically, when comparing the ITT and original data analyses, although the general improvement trends were consistent, the statistical significance of some group‐by‐time interaction effects was attenuated in the original sample. This discrepancy is likely attributable to the reduced statistical power resulting from the smaller sample size in the original data set, highlighting the importance of the ITT approach. Future studies with larger cohorts are needed to replicate these findings and to help determine which patient phenotypes respond best. Third, the absence of a control group receiving only Hi‐tACS prevents clear attribution of effects to either neuromodulation or rehabilitation alone. Although the combined intervention reflects real‐world clinical settings, it restricts causal interpretation.

## 5 Conclusion

In conclusion, this trial is the first randomized controlled trial to evaluate the effect of Hi‐tACS as an add‐on to MIRT in PD. The 24‐week observation period provides preliminary indications on the potential durability of QoL and non‐motor effects. Future research should expand the sample size and explore the optimal timing and sequencing of Hi‐tACS relative to MIRT. In addition, multimodal neuroimaging and electrophysiological data should continue to be utilized to explore the neural mechanisms underlying treatment effects, thereby providing a basis for optimizing future neuromodulation strategies.

## Author Contributions

Bo‐yan Fang and Ting‐ting Hou, had full access to all the data in the study and took responsibility for the integrity of the data and the accuracy of the data analysis. Concept and design: Bo‐yan Fang, Bastiaan R. Bloem, Ting‐ting Hou, Hong‐yu Zhang. Methodological guidance: Xiao‐yan Yan. Data collection: Ting‐ting Hou, Hong‐yu Zhang. Clinical management: Ting‐ting Hou, Hong‐yu Zhang, Ke‐ke Chen, Zhao‐hui Jin, Tian Zhang, Lin Qi, Cui‐ping Xue, Qiao‐xia Zhen, Zhen‐zhen Li, Hong‐jiao Yan, Yi Zhen, Xia An, Jia Du, Yuan Su, Cui Liu, Qi‐ping Wen, Bo‐yan Fang. Statistical analysis: Ting‐ting Hou. Drafting of the manuscript: Ting‐ting Hou. Critical revision of the manuscript for important intellectual content: Bo‐yan Fang, Bastiaan R. Bloem. Obtained funding: Bo‐yan Fang, Zhao‐hui Jin. Administrative, technical, or material support: Bo‐yan Fang, Zhao‐hui Jin. Supervision: Bo‐yan Fang.

## Funding

This work was supported by National Key Research and Development Program of China (2022YFC3602603). Beijing Shijingshan District Medical Key Discipline Research Program (2023002).

## Disclosure

Additional Supporting Information can be found online in the Supporting Information section.

## Ethics Statement

The protocol was approved by the Ethics Committee of Beijing Rehabilitation Hospital, Capital Medical University (Ethical review No. 2023kky‐064).

## Conflicts of Interest

All authors declare no Conflicts of Interest.

## Supporting information


**Table S1:** Baseline demographics of the original sample.
**Table S2:**. Between‐group differences in the primary outcome (PDQ‐39 scores) in the ITT sample.
**Table S3:**. Between‐group differences in the primary outcome (PDQ‐39 scores) in the original sample.
**Table S4:**. Between‐group differences in secondary outcomes (motor and non‐motor symptoms) in the ITT sample.
**Table S5:**. Between‐group differences in secondary outcomes (motor and non‐motor symptoms) in the original sample.
**Table S6:**. Summary of the most common non‐serious adverse events and duration experienced by participants in both groups.

**Supinfo S1**. Original study protocol, detailing the rationale, study design, intervention procedures, and statistical analysis methods.

## Data Availability

The data that support the findings of this study are available from the corresponding author upon reasonable request.
