# Supplementary material for: High‐Intensity Alternating Current Stimulation as an Add‐On to Multidisciplinary Intensive Rehabilitation for Parkinson's Disease: A Randomized Controlled Trial
Source: CNS Neurosci Ther. 2026 Apr 27;32(4):e70909. doi: 10.1002/cns.70909 (PMC13121914; doi:10.1002/cns.70909)
Supplement: Supplementary file 1 — Table S1: Baseline demographics of the original sample. Table S2:. Between‐group differences in the primary outcome (PDQ‐39 scores) in the ITT sample. Table S3:. Between‐group differences in the primary outcome (PDQ‐39 scores) in the original sample. Table S4:. Between‐group differences in secondary outcomes (motor and non‐motor symptoms) in the ITT sample. Table S5:. Between‐group differences in secondary outcomes (motor and non‐motor symptoms) in the original sample. Table S6:. Summary of the most common non‐serious adverse events and duration experienced by participants in both groups. [file CNS-32-e70909-s001.docx]

**Supinfo S1**

**ORIGINAL STUDY PROTOCOL**

**Project title**

High-Intensity Alternating Current Stimulation as an Add-On to Multidisciplinary Intensive Rehabilitation for Parkinson’s Disease: A Randomised Controlled Trial

**Abstract**

**Background/Objective:** Parkinson’s disease (PD) profoundly affects quality of life (QoL) through persistent motor and non-motor symptoms, and current rehabilitation approaches often fail to produce sustained benefits. Multidisciplinary intensive rehabilitation therapy (MIRT) can generate short-term improvements, but its effects typically decline within several months. High-intensity transcranial alternating current stimulation (Hi-tACS) has emerged as a neuromodulation technique capable of engaging deeper neural circuits, yet its therapeutic value in PD remains insufficiently explored. This study aims to determine whether combining Hi-tACS with MIRT produces more durable improvements in QoL compared with MIRT alone in individuals with PD.

**Participants:** A total of 60 individuals diagnosed with idiopathic PD, classified within Hoehn and Yahr stages 1 to 3 and aged between 45 and 70 years, were enrolled in the trial.

**Design:** This was a double-blind, randomized, placebo-controlled clinical study, with follow-up assessments at 4, 12, and 24 weeks post-intervention.

**Interventions:** All participants received 10 consecutive days of MIRT, which included daily sessions of physical, gait, aerobic, and speech training. The intervention group additionally underwent twice-daily Hi-tACS stimulation (77.5 Hz, 15 mA, 40 minutes per session), while the control group received sham stimulation of identical duration and placement.

**Primary outcomes:** The main efficacy measure was the change in scores on the 39-item Parkinson’s Disease Questionnaire (PDQ-39) from baseline (T0) to weeks 4 (T2), 12 (T3), and 24 (T4) in the medication “off” state.

**Secondary outcomes:** Secondary assessments included comprehensive evaluations of motor and non-motor symptoms.

**Impact of the project:** This protocol evaluates an innovative combined neuromodulation–rehabilitation strategy designed to enhance the durability of functional and psychosocial outcomes in PD. If effective, the integration of Hi-tACS with MIRT could provide a scalable approach to sustaining QoL. The findings may inform future therapeutic frameworks and guide the development of closed-loop neurorehabilitation paradigms for PD.

**Trial registration:** Chinese Clinical Trial Registry ChiCTR2300071969. Registered on 30 May 2023.

**TABLE OF CONTENTS**

1. INTRODUCTION AND RATIONALE ........................................................................................................................................................................ 5

2. OBJECTIVES ................................................................................................................................................................................................................ 6

2.1 Primary objective ....................................................................................................................................................................................................... 6

2.2 Secondary objective ................................................................................................................................................................................................... 6

3. STUDY DESIGN ........................................................................................................................................................................................................... 6

4. STUDY POPULATION ................................................................................................................................................................................................. 6

4.1 Recruitment .............................................................................................................................................................................................................. 6

4.2 Inclusion criteria ........................................................................................................................................................................................................7

4.3 Exclusion criteria .......................................................................................................................................................................................................7

4.4 Dropping criteria ........................................................................................................................................................................................................8

4.5 Sample size calculation ............................................................................................................................................................................................ 8

5. TREATMENT OF SUBJECTS .......................................................................................................................................................................................8

5.1 High-intensity Transcranial Alternating Current Stimulation ...................................................................................................................................8

5.2 Multidisciplinary Intensive Rehabilitation Therapy procedure .................................................................................................................................9

6. SAFETY and ADVERSE EVENT REPORTING ..........................................................................................................................................................9

7. METHODS ....................................................................................................................................................................................................................10

7.1 Study procedures .....................................................................................................................................................................................................10

7.2 Study setting ............................................................................................................................................................................................................10

7.3 Data collection and management .............................................................................................................................................................................10

7.3.1 Data collection ....................................................................................................................................................................................................10

7.3.2 Data management ...............................................................................................................................................................................................11

7.3.3 Data Confidentiality............................................................................................................................................................................................11

7.4 Study outcomes .......................................................................................................................................................................................................11

7.4.1 Primary outcomes ...............................................................................................................................................................................................12

7.4.2 Secondary outcomes ...........................................................................................................................................................................................12

7.5 Randomisation, blinding and treatment allocation ..................................................................................................................................................12

7.6 Ethical considerations…...........................................................................................................................................................................................13

7.7 Intervention adherence.............................................................................................................................................................................................13

7.8 Relevant concomitant care.......................................................................................................................................................................................13

8. STATISTICAL ANALYSIS .........................................................................................................................................................................................13

8.1 Primary outcomes.....................................................................................................................................................................................................14

8.2 Secondary outcomes ................................................................................................................................................................................................14

9. STUDY MONITORING................................................................................................................................................................................................14

10. REFERENCES.............................................................................................................................................................................................................15

1. INTRODUCTION AND RATIONALE

Parkinson's disease (PD) presents with core motor manifestations (bradykinesia, rigidity, postural instability, resting tremor) and debilitating non-motor features, including cognitive dysfunction and affective disorders, among others,^1, 2^ thereby reducing patients' overall quality of life (QoL).^3^ While pharmacological treatment remains the cornerstone of symptom control, it is often insufficient to halt disease progression or provide meaningful relief from non-motor symptoms such as depression, anxiety, cognitive decline, and fatigue. In recent years, non-pharmacological interventions, particularly rehabilitation therapies, have shown potential to improve functional outcomes and QoL.^4, 5^ However, conventional rehabilitation strategies typically require extended durations (often ≥6 months) and offer limited long-term efficacy, especially when it comes to maintaining benefits beyond the active treatment phase.

Multidisciplinary Intensive Rehabilitation Therapy (MIRT) has emerged as a condensed and targeted alternative to traditional rehabilitation, combining intensive motor, cognitive, and speech therapy over a shorter period.^6^ Prior studies, including our own, have demonstrated significant short-term motor improvements following MIRT. However, these gains often diminish within 3 months,^7^ highlighting a persistent challenge in sustaining therapeutic benefits in PD rehabilitation.

Transcranial alternating current stimulation (tACS) represents a promising neuromodulation technique for PD rehabilitation.^8^ The extracellular application of alternating current modulates neuronal membrane potentials in a phase-dependent manner, influencing neuronal firing rates and amplifying effects through network-level interactions.^9, 10^ Accumulating evidence suggests that tACS can regulate cortical oscillations and enhance motor and cognitive functions in people with Parkinson’s disease (PWP).^11-14^ However, most studies to date have applied low-intensity stimulation (<2 mA) due to safety concerns. While these trials reported some improvements in bradykinesia, tremor, and cognition, the limited energy and stimulation scope often led to inconsistent or transient benefits.^12, 15^ Recent advances highlight the potential of high-intensity tACS (Hi-tACS; 77.5 Hz, 15 mA), delivered via forehead–mastoid electrodes, to modulate deep brain structures and influence neurochemical activity, including beta-endorphin and neurotransmitter levels in the cerebrospinal fluid, brainstem, and cortex. Hi-tACS has shown safety and efficacy in conditions such as chronic insomnia, refractory epilepsy, and mood disorders.^16, 17^ Despite its promise, Hi-tACS has not been systematically investigated in PD.

Only one Hi-tACS study on PWP did not show significant improvements in motor and psychological symptoms compared to placebo intervention.^18^ We have identified several potential reasons for these findings. Firstly, the previous trial only administered tACS treatments 10 times per patient, which may have resulted in an insufficient cumulative dosage that contributed to the ultimately negative outcome. In our experiment, each patient received 2 daily sessions of tACS over a period of 10 days, totaling 20 treatment sessions, thereby increasing the dosage in order to achieve more favorable results. Additionally, the prior investigation had a limited sample size of only 23 participants for their simple tACS study, potentially influencing the negative outcomes observed. To address this limitation and provide stronger data support for our trial, we aimed to recruit 60 participants and expand the sample size accordingly. Critically, the previous trials have primarily focused on the administration of tACS alone, whereas our trial integrates tACS with MIRT based on a concept of “cortical prehabilitation”, which refers to the proactive modulation of dysfunctional neural networks before or during functional training, with the aim of improving rehabilitation receptivity and outcomes.

To our knowledge, no randomized controlled trials have yet assessed the combined use of Hi-tACS and MIRT in PD. This study aims to evaluate whether adding Hi-tACS to a standardized MIRT program can produce more durable and multidimensional improvements in individuals with idiopathic PD compared to MIRT alone.

2. OBJECTIVES

2.1 Primary objective

To investigate the impact of Hi-tACS on improving the QoL in PWP.

2.2 Secondary objective

• To investigate the effect of Hi-tACS on motor and non-motor symptoms in PWP.

3. STUDY DESIGN

This will be a double blind, placebo-controlled randomized clinical trial.

4. STUDY POPULATION

4.1 Recruitment

Recruitment commenced on June 1, 2023, and concluded on December 31, 2023, or upon reaching the required number of patients, whichever transpired first. If there is an insufficient number of registrations before the deadline, we will submit an extension application. Participant recruitment encompassed two strategies: The initial step involves referring patients who visit the outpatient clinic at Beijing Rehabilitation Hospital to project researchers for recruitment purposes. The recruitment notices were additionally disseminated through the hospital’s official website and various other online platforms, facilitating interested patients to schedule appointments online conveniently. Patients with confirmed appointments underwent initial telephone screening conducted by a rehabilitation physician to assess their eligibility prior to undergoing on-site evaluation at the outpatient clinic. Ultimately, eligible participants who provided written informed consent were randomized and received treatment.

4.2 Inclusion criteria

In order to be eligible to participate in this study a subject must meet the following criteria:

• Conformity with the diagnostic criteria of idiopathic PD according to the 2015 Movement Disorder Society (MDS) criteria.^19^

• Patients that are classified as 1–3 according to the Hoehn and Yahr scale.^20^

• Age of 45–70 years and primary school education level and above.

• The drug effect was stable for more than 2 weeks without adjustment, and no DBS surgery was performed.

• Those who can walk without assistance (including assistive devices) and need no help from others in daily life.

• Those who voluntarily sign the informed consent form and confirm that they will be able to complete the treatment.

4.3 Exclusion criteria

A potential subject who meets any of the following criteria will be excluded from participation in this study:

• The diagnosis is unclear or suspected of Parkinson’s syndrome (such as vascular, drug-induced, and post-infection Parkinson’s syndrome), multiple system atrophy, progressive supranuclear palsy, etc..

• Patients with cognitive, motor, speech, and other impairments caused by other nervous system diseases.

• A history of epilepsy or other contraindications to tACS.

• Subjects who are unwilling or unable to participate in MRI examinations (such as those with claustrophobia or non-MRI-compatible implants in the body).

• Serious concomitant diseases such as heart, lung, liver, and kidney insufficiency.

• Those who are participating in other clinical trials or are going to undergo DBS surgery or participate in other clinical trials in the next 12 months.

• Patients who are unable to cooperate in completing the treatment or examination.

• Patients who are not willing to sign the informed consent form.

4.4 Dropping criteria

• Patients who revoke their informed consent.

• Patients who discontinued treatment due to various factors.

4.5 Sample size calculation

PASS11 software was used to calculate the sample size. According to the previous literature,^21, 22^ which includes the results of PDQ-39 in individuals with PD, and in combination with this experiment, we designed a ratio of 1:1 between the tACS + MIRT group and the Sham-tACS + MIRT group. The statistical power was set at 0.9, with a significance level (α) of 0.05. Based on these parameters, the required sample size for each group was calculated as 27. Considering an attrition rate of 10%, it was determined that each group would need a total of 30 patients, resulting in a combined total of 60 patients.

5. TREATMENT OF SUBJECTS

The application of Hi-tACS as a non-invasive brain stimulation (NIBS) technique in clinical treatment is well-established. In this study, the inclusion of sham-Hi-tACS + MIRT in the control group served to elicit a placebo effect, with the only discernible distinction between the Hi-tACS + MIRT group and sham-Hi-tACS + MIRT being the presence or absence of electrical current.

After the initial baseline assessment (days 1–2), patients will be administered Hi-tACS + MITR or sham Hi-tACS + MIRT for a consecutive period of 10 days (days 3–12).

5.1 High-intensity Transcranial Alternating Current Stimulation

During each treatment session, patients will receive Hi-tACS or sham Hi-tACS stimulation for 40 min twice a day. The treatment duration was standardized for each day (40 min per session, twice daily for 10 days, totaling 20 sessions). Patients will receive a 10-day MIRT program based on Hi-tACS treatment, which includes four distinct rehabilitation exercises per day, with each session lasting 30–60 minutes. Participants were comfortably seated on reclining chairs to receive FDA-approved Hi-tACS from Nexalin Technology, Inc. The administration of Hi-tACS was carried out by trained medical professionals following standardized instructions. This trial utilized two Hi-tACS devices, one sham and one active, which were identical in terms of size, color, appearance, weight, and odor. Each participant was consistently assigned to the same device throughout the entire intervention. Patients were instructed to consume water prior to the intervention, maintain a state of relaxation or even sleep, and minimize communication with medical personnel. Three Nexalin conductive electrodes were positioned above the head according to the 10/20 international placement system, with a 4.45 × 9.53 cm electrode placed on the forehead corresponding to Fpz, Fp1, and Fp2. Two 3.18 × 3.81 cm electrodes were placed on each side in the mastoid region. The Hi-tACS stimulation waveform consisted of ramp-up and ramp-down periods lasting for 180 s and 12 s, respectively. It was a square wave with an average amplitude of 15 mA and was equally distributed from the frontal region to the mastoid areas (amplitudes are reported as zero-to-peak). All participants received a total of twenty sessions involving either true or sham stimulation at a frequency of 77.5 Hz and intensity of 15 mA, respectively. Sham Hi-tACS had no active stimulation.

5.2 Multidisciplinary Intensive Rehabilitation Therapy procedure

I. Physical therapy. It involves warm-up activities followed by active and passive stretching and flexibility training. The physical therapist will conduct sessions in groups of four for a duration of 40 minutes.

II. Gait balance training. C-MiLL (Motek, Amsterdam/ Culemborg, Netherlands) and Balance Tutor (Meditouch, Netanya, Israel) will be utilized to enhance balance and gait. Patients will undergo 30-minute training sessions once per day.

III. Aerobic training. Patients will engage in a 30-minute aerobic workout using an upper and lower limb trainer (T5XR; Nustep, Ann Arbor, MI, USA).

IV. Speech therapy. The speech therapist will conduct one-hour group sessions of 4 patients.

Each therapeutic module was conducted by the same certified therapists to ensure consistency in therapeutic outcomes. Throughout the trial, we will have the option to modify parameters or discontinue treatment entirely in response to any adverse events that may arise. Physicians and therapists will document any such events in writing within the Electronic Data Capture (EDC) system for review by the study team. Additionally, falls, injuries, and other harmful incidents must be reported by regulations set forth by Beijing Rehabilitation Hospital affiliated with Capital Medical University.

6. SAFETY and ADVERSE EVENT REPORTING

The Hi-tACS procedure is considered safe, with no apparent short- or long-term harm observed. Potential low-grade adverse events may include seizures, headaches, site irritation, pain, etc. However, post-treatment monitoring will be conducted through interviews about conditions such as discomfort, pain, or injury to assess any side effects. All serious adverse events (SAEs) must be promptly reported within 24 h of occurrence to the principal investigator, ethics committee, and data and safety monitoring board (DSMB). Following thorough discussion and evaluation of these issues by the DSMB and principal investigators, they will have discretionary authority to determine whether discontinuation of the trial is necessary

7. METHODS

7.1 Study procedures

The present protocol follows the Standard Protocol Items Recommendations for Interventional Trials (SPIRIT) guidelines and fulfills the SPIRIT checklist. We hypothesize that Hi-tACS combined with MIRT is superior in terms of efficiency and effectiveness compared to MIRT alone for treating PD. Each patient’s participation in the study will last for 26 weeks, including baseline evaluation (T0), a 10-day intervention period, and assessments at the end of intervention (T1), as well as 4 weeks (T2), 12 weeks (T3), and 24 weeks (T4) post-intervention.

7.2 Study setting

This study will be conducted at a single site: Beijing Rehabilitation Hospital, Capital Medical University. The Department of Scientific Research Management of Beijing Rehabilitation Hospital was responsible for overseeing the trial.

7.3 Data collection and management

7.3.1 Data collection

The following data will be collected at baseline: gender, age, occupation, educational level, current medication status, and detailed physical and neurological examinations. Inclusion and exclusion criteria will be evaluated. PDQ-39 assessments will be conducted at T0, T2, T3, and T4. Motor and non-motor outcomes will be evaluated at T0, T1, T2, T3, and T4. (Corrections: due to the need for professional in-person evaluation to ensure the quality of some motor assessments, a substantial amount of follow-up motor data was missing, as many participants were unable to return for face-to-face visits. As a result, motor outcomes were analyzed only at T0 and T1. In contrast, non-motor outcomes were collected at T0, T2, T3, and T4, as they could be reliably assessed via in-person, telephone, or video follow-ups. T1 was excluded from non-motor analysis since most non-motor scales reflect the patient's status over the past month, making T1 an inappropriate time point for evaluation). MRI and EEG scans will be evaluated at T0 and T1. Levodopa equivalent daily dose (LEDD) data will be collected at T0, T1, T2, T3, and T4. All these assessments will be conducted in a double blind manner by raters who were unaware of the treatment administered to the patients. At T0 and T1, various safety indicators, including blood routine, urine routine, stool routine, liver and kidney function tests, electrocardiogram readings, and other relevant parameters will be evaluated. Any adverse events occurring during the study will be documented. Participants will be asked for their consent regarding the use of their data if they choose to withdraw from the trial. Additionally, participants must obtain permission from the research team to share their pertinent information with university personnel involved in the study or relevant regulatory authorities. Before commencing the study, investigators will undergo standardized training on data collection strategies as well as guidelines for utilizing different scales to assess outcomes. Throughout the trial period, professionally trained researchers will collect data to ensure impartiality.

To eliminate the potential influence of dopaminergic medication, all clinical assessments were conducted during the medication-off state (≥12 hours after levodopa withdrawal). Only the “off” condition was used for all analyses and outcome evaluations. Follow-up assessments were completed via an online system or in person at the outpatient clinic.

7.3.2 Data management

Based on the clinical research data platform of Parkinson Medical Center, Beijing Rehabilitation Hospital, the EDC system will be utilized for data entry and management. The task of data entry will be performed by trained data collectors. Participant information will be securely stored in the EDC system, accessible to researchers through password authentication. Rigorous checks will ensure the completeness of case report forms and consistency between coding and subjects screened for enrollment. Data validation procedures will be implemented upon entry into the EDC system, with subsequent review of original cases based on these results. The final data set will be exported as an Excel spreadsheet (Microsoft, Redmond, WA, USA). Boyan Fang is responsible for conducting an interim analysis while making a final decision regarding trial suspension based on these interim results. Access to the finalized trial dataset will only be granted to authorized researchers.

7.3.3 Data Confidentiality

The privacy of all participants will be safeguarded by assigning their information a unique trial identification code, and the study data will be securely stored in a password-protected file accessible only to the data manager of the research team.

7.4 Study outcomes

7.4.1 Primary outcomes

The primary outcome indicator will be the PDQ-39, which assesses changes in QoL from T0 to T2, T3, and T4. The PDQ-39 consists of 39 items that are categorized into eight domains: mobility, activities of daily living, emotional well-being, stigma, social support, cognitions, communication, and bodily discomfor.^23^ Patients were instructed to indicate the frequency of occurrence for each corresponding event within the preceding month. The outcome of the scale encompasses a comprehensive index score, eight sub-core scores, and a weighted percentage reflecting problem severity. Higher scores (ranging from 0 to 100) indicate a greater burden on QoL, with − 4.72 and + 4.22 serving as clinically significant thresholds for detecting improvements or deteriorations in QoL among individuals with PD.^24^ The PDQ-39 has been translated and validated in multiple languages and cultural settings, making it a recommended tool by the Movement Disorders Society due to its proven effectiveness and stability.^25^

7.4.2 Secondary outcomes

Motor function will be assessed using instruments such as the Movement Disorder Society-Unified Parkinson’s Disease Rating Scale Part III (MDS-UPDRS III), the Modified Parkinson Activity Scale (M-PAS), the Berg Balance Scale (BBS), and the Timed Up and Go Test (TUG).

Non-motor symptoms will be evaluated using the Non-Motor Symptoms Scale (NMSS), the Geriatric Depression Scale (GDS), Hamilton Anxiety Scale (HAMA), Hamilton Depression Scale (HAMD), and the Modified Apathy Estimate Scale (MAES), etc.

We will list all positive results.

7.5 Randomisation, blinding and treatment allocation

The randomization of patients in a 1:1 ratio will be conducted by an independent researcher who is not involved in the assessments or stimulation. This researcher will be solely responsible for dispensing randomization numbers to patients in the order of enrollment throughout the trial. We will utilize the complete randomization function of SPSS 27.0 statistical software (IBM, Chicago, IL, USA) to generate a table of random numbers, and the SPSS Visual Binning function has been integrated into its system. The process of randomization will be overseen by a blinded worker from DMC who will have exclusive authority over managing the electronic coding for assigning individuals. The process will ensure complete automation without any human intervention and will remain entirely undisclosed to both study investigators and prospective participants until the assignment of study groups. This document will be entrusted to Beijing Rehabilitation Hospital, Capital Medical University for secure storage.

The coding group for the assignment concealment process will be placed in a sealed envelope, which will be labeled with each participant’s code and securely held by the staff responsible for randomization. The envelopes will only be opened during Hi-tACS or sham-Hi-tACS sessions. To ensure proper blinding, participants will receive a password that will remain concealed during allocation by an independent staff member responsible for randomization. The Hi-tACS experimenter, who administers the intervention, will also remain unaware of the group assignments. Both the Hi-tACS and sham-Hi-tACS devices will have identical appearances and be marked as either A or B. Additionally, a DMC staff member responsible for randomization will inform the Hi-tACS experimenter to use either device A or B. The sealed envelopes will be opened and resealed by the staff responsible for randomization before intervention. Subsequently, the Hi-tACS experimenter will be informed of the code.

Both participants and clinic staff (including outcome assessors, caregivers, nurses, physical therapists, and statistical analysts) will remain blinded to group allocation until the completion of the study. Only the randomized investigators will have knowledge of the group assignments but will not disclose them to the patients. To ensure double-blinding, patients will be instructed not to discuss their treatment group with other patients or staff members. The disclosure of whether the intervention is Hi-tACS or sham-Hi-tACS will be withheld throughout the study.

When encountering serious adverse events that necessitate immediate unblinding, the scientific research management department head, data management department project leader, and statistician will jointly perform the unblinding process and meticulously document it. Unblinding will solely disclose the treatment received by a patient through a randomized number, without impacting the blinding of other participants. It will not be involved in efficacy analyses, but will be included in safety analyses

7.6 Ethical considerations

The participation is on a voluntary basis and participants could withdraw from the study at any time point. This protocol is stated in compliance with the Declaration of Helsinki.

7.7 Intervention adherence

The participants will receive complimentary MIRT treatment, EEG, and MRI examinations to promote adherence to the interventions.

7.8 Relevant concomitant care

The concomitant therapies involve the administration of medications such as levodopa and benserazide, in addition to measures aimed at preventing complications. All other non-invasive or invasive brain stimulation interventions, including transcranial direct current stimulation or deep brain stimulation, will be strictly prohibited. Provisions for ancillary and post-trial care are not relevant to the study.

8. STATISTICAL ANALYSIS

All analyses will primarily follow the intention-to-treat principle, with per-protocol analysis conducted as a secondary approach if participants are unable to complete the intervention due to issues such as adverse effects or poor compliance. Missing data will be addressed using multiple imputation, and sensitivity analyses will be considered in cases of high attrition or imbalanced follow-up losses. Statistical analyses will be conducted using SPSS 27.0. Continuous variables will be assessed for normality using the Shapiro–Wilk test and presented accordingly. Categorical data will be summarized as counts and percentages. A two-tailed P value < 0.05 will be considered statistically significant.

8.1 Primary outcomes

To evaluate changes across multiple timepoints (T0, T2, T3, T4) in PDQ-39 scores, generalized estimating equation (GEE) models will be employed. Time will be treated as a within-subject factor, and group as a between-subject factor.

8.2 Secondary outcomes

Between-group differences in motor function changes from baseline to post-intervention (T1–T0) will be assessed using independent samples t-tests for normally distributed data or the Mann–Whitney U test for non-parametric data, as appropriate.

To evaluate changes across multiple timepoints (T0, T2, T3, T4) in non-motor function, generalized estimating equation (GEE) models will be employed. Time will be treated as a within-subject factor, and group as a between-subject factor.

9. STUDY MONITORING

We have established an independent DSMB for this study, which is not sponsored by any particular organization. The committee comprises a rehabilitation specialist, a neurologist, and a statistician who will monitor adherence to the trial design and standard guidelines.

The DSMB will oversee the trial design and adherence to standard guidelines. This study, conducted at Beijing Rehabilitation Hospital, is a single-center investigation managed by the DSMB. A committee of rehabilitation specialists will be responsible for controlling the study protocol, while a neurologist will evaluate, manage, and classify all adverse events (AEs) that occur. Additionally, a statistician will review the data for safety purposes. It is important to note that DSMB members are independent of each other with no conflicts of interest.

10. REFERENCES

**1.** Jankovic J, Tan EK. Parkinson's disease: etiopathogenesis and treatment. *J Neurol Neurosurg Psychiatry.* Aug 2020;91(8):795-808.

**2.** Leite Silva ABR, Goncalves de Oliveira RW, Diogenes GP, et al. Premotor, nonmotor and motor symptoms of Parkinson's Disease: A new clinical state of the art. *Ageing Res Rev.* Feb 2023;84:101834.

**3.** Collaborators GBDPsD. Global, regional, and national burden of Parkinson's disease, 1990-2016: a systematic analysis for the Global Burden of Disease Study 2016. *Lancet Neurol.* Nov 2018;17(11):939-953.

**4.** van Wamelen DJ, Leta V, Chaudhuri KR, Jenner P. Future Directions for Developing Non-dopaminergic Strategies for the Treatment of Parkinson's Disease. *Curr Neuropharmacol.* 2024;22(10):1606-1620.

**5.** Wamelen DJV, Rukavina K, Podlewska AM, Chaudhuri KR. Advances in the Pharmacological and Non-pharmacological Management of Non-motor Symptoms in Parkinson's Disease: An Update Since 2017. *Curr Neuropharmacol.* 2023;21(8):1786-1805.

**6.** Reid M, Mitchell SD, Mitchell KM, Sidiropoulos C. Efficacy of a 5-day, intensive, multidisciplinary, outpatient physical and occupational therapy protocol in the treatment of functional movement disorders: A retrospective study. *J Neurol Sci.* Dec 15 2022;443:120461.

**7.** Chen KK, Jin ZH, Gao L, et al. Efficacy of short-term multidisciplinary intensive rehabilitation in patients with different Parkinson's disease motor subtypes: a prospective pilot study with 3-month follow-up. *Neural Regen Res.* Jul 2021;16(7):1336-1343.

**8.** Elyamany O, Leicht G, Herrmann CS, Mulert C. Transcranial alternating current stimulation (tACS): from basic mechanisms towards first applications in psychiatry. *Eur Arch Psychiatry Clin Neurosci.* Feb 2021;271(1):135-156.

**9.** Krause MR, Vieira PG, Csorba BA, Pilly PK, Pack CC. Transcranial alternating current stimulation entrains single-neuron activity in the primate brain. *Proc Natl Acad Sci U S A.* Mar 19 2019;116(12):5747-5755.

**10.** Reato D, Rahman A, Bikson M, Parra LC. Effects of weak transcranial alternating current stimulation on brain activity-a review of known mechanisms from animal studies. *Front Hum Neurosci.* Oct 23 2013;7:687.

**11.** Brak IV, Filimonova E, Zakhariya O, Khasanov R, Stepanyan I. Transcranial Current Stimulation as a Tool of Neuromodulation of Cognitive Functions in Parkinson's Disease. *Front Neurosci.* 2022;16:781488.

**12.** Guerra A, Colella D, Giangrosso M, et al. Driving motor cortex oscillations modulates bradykinesia in Parkinson's disease. *Brain.* Mar 29 2022;145(1):224-236.

**13.** Kim J, Kim H, Jeong H, Roh D, Kim DH. tACS as a promising therapeutic option for improving cognitive function in mild cognitive impairment: A direct comparison between tACS and tDCS. *J Psychiatr Res.* Sep 2021;141:248-256.

**14.** Madrid J, Benninger DH. Non-invasive brain stimulation for Parkinson's disease: Clinical evidence, latest concepts and future goals: A systematic review. *J Neurosci Methods.* Jan 1 2021;347:108957.

**15.** Antal A, Alekseichuk I, Bikson M, et al. Low intensity transcranial electric stimulation: Safety, ethical, legal regulatory and application guidelines. *Clin Neurophysiol.* Sep 2017;128(9):1774-1809.

**16.** Wang H, Wang K, Xue Q, et al. Transcranial alternating current stimulation for treating depression: a randomized controlled trial. *Brain.* Mar 29 2022;145(1):83-91.

**17.** Wang HX, Wang L, Zhang WR, et al. Effect of Transcranial Alternating Current Stimulation for the Treatment of Chronic Insomnia: A Randomized, Double-Blind, Parallel-Group, Placebo-Controlled Clinical Trial. *Psychother Psychosom.* 2020;89(1):38-47.

**18.** Shill HA, Obradov S, Katsnelson Y, Pizinger R. A randomized, double-blind trial of transcranial electrostimulation in early Parkinson's disease. *Mov Disord.* Jul 2011;26(8):1477-1480.

**19.** Postuma RB, Berg D, Stern M, et al. MDS clinical diagnostic criteria for Parkinson's disease. *Mov Disord.* Oct 2015;30(12):1591-1601.

**20.** Goetz CG, Poewe W, Rascol O, et al. Movement Disorder Society Task Force report on the Hoehn and Yahr staging scale: status and recommendations. *Mov Disord.* Sep 2004;19(9):1020-1028.

**21.** Li J, Mi TM, Zhu BF, et al. High-frequency repetitive transcranial magnetic stimulation over the primary motor cortex relieves musculoskeletal pain in patients with Parkinson's disease: A randomized controlled trial. *Parkinsonism Relat Disord.* Nov 2020;80:113-119.

**22.** Meng D, Jin Z, Chen K, et al. Quality of life predicts rehabilitation prognosis in Parkinson's disease patients: Factors influence rehabilitation prognosis: Factors influence rehabilitation prognosis. *Brain Behav.* May 2022;12(5):e2579.

**23.** Jenkinson C, Fitzpatrick R, Peto V, Greenhall R, Hyman N. The Parkinson's Disease Questionnaire (PDQ-39): development and validation of a Parkinson's disease summary index score. *Age Ageing.* Sep 1997;26(5):353-357.

**24.** Horvath K, Aschermann Z, Kovacs M, et al. Changes in Quality of Life in Parkinson's Disease: How Large Must They Be to Be Relevant? *Neuroepidemiology.* 2017;48(1-2):1-8.

**25.** Martinez-Martin P, Jeukens-Visser M, Lyons KE, et al. Health-related quality-of-life scales in Parkinson's disease: critique and recommendations. *Mov Disord.* Nov 2011;26(13):2371-2380.

**26.** Keus SH, Nieuwboer A, Bloem BR, Borm GF, Munneke M. Clinimetric analyses of the Modified Parkinson Activity Scale. *Parkinsonism Relat Disord.* May 2009;15(4):263-269.

**SUPPLEMENTARY TABLE**

**Table S1. Baseline demographics in the** **original sample**

| Characteristics | All (n = 56) | Hi-tACS+MIRT (n=27) | sham-Hi-tACS+MIRT (n=29) |
| --- | --- | --- | --- |
| Age, median (IQR), y | 64 (57, 68) | 65 (60, 68) | 61 (55.5, 68) |
| Sex, n (%) | | | |
| Male | 23 (41.4) | 11 (40.7) | 12 (41.4) |
| Female | 33 (58.9) | 16 (59.3) | 17 (58.6) |
| Affected side, n (%) | | | |
| Left | 22 (39.3) | 13 (48.1) | 9 (31.0) |
| Right | 34 (60.7) | 14 (51.9) | 20 (69.0) |
| Hoehn & Yahr (on^a^) , n (%) | | | |
| 1.0 | 2 (3.6) | 2 (7.4) | 0 (0) |
| 1.5 | 14 (25) | 8 (29.6) | 6 (20.7) |
| 2.0 | 28 (50.0) | 14 (51.9) | 14 (48.3) |
| 2.5 | 5 (8.9) | 1 (3.7) | 4 (13.8) |
| 3.0 | 7 (12.5) | 2 (7.4) | 5 (17.2) |
| Hoehn & Yahr (off^b^) , n (%) | | | |
| 1.0 | 0 (0) | 0 (0) | 0 (0) |
| 1.5 | 11 (19.6) | 7 (25.9) | 4 (13.8) |
| 2.0 | 27 (48.2) | 14 (51.9) | 13 (44.8) |
| 2.5 | 8 (14.3) | 4 (14.8) | 4 (13.8) |
| 3.0 | 10 (17.9) | 2 (7.4) | 8 (27.6) |
| Disease Duration, y | 7.2 ± 3.1 | 6.9 ± 2.5 | 7.5 ± 3.6 |
| Treatment duration, y | 5.9 ± 3.1 | 5.4 ± 2.7 | 6.3 ± 3.4 |
| Years of education, median (IQR), y | 12 (12, 15.8) | 13 (11, 16) | 12 (12, 15.5) |
| LEDD | 555.62 ± 216.66 | 551.32 ± 237.60 | 559.62 ± 199.37 |

IQR=interquartile range; LEDD=Levodopa equivalent daily dose.

^a^“On” represents “on state” of levodopa treatment (1-2 hours post-levodopa).

^b^“Off” represents for “off state” of levodopa treatment (≥12 hours post-levodopa).

**Table S2. Differences between two groups in primary outcomes in the ITT sample**

| Outcomes | Timepoint | Hi-tACS+MIRT  (n=30) | sham-Hi-tACS+MIRT  (n=30) | Effect size  (95% CI) | *P_FDR_* ^a^ | *P*-group^b^ | *P*-group*time^c^ |
| --- | --- | --- | --- | --- | --- | --- | --- |
| PDQ-39, median (IQR) | T0 | 18.59(10.25,28.04) | 20.83(18.26,30.92) | 0.39(-0.13,0.90) |  | < 0.001^*^ | 0.008^*^ |
|  | T2 | 14.42(6.41,19.87) | 20.83(15.06,30.92) | 0.68(0.14,1.21) | 0.009^*^ |  |  |
|  | T3 | 14.42(5.76,20.67) | 21.47(11.85,31.04) | 1.43(0.80,2.05) | 0.003^*^ |  |  |
|  | T4 | 9.61(5.60,19.39) | 28.38(17.94,35.41) | 1.26(0.65,1.85) | <0.001^*^ |  |  |

IQR = interquartile range; PDQ-39 = the 39-item Parkinson’s Disease Questionnaire

^a^Statistical differences between groups comparing at the same time point with FDR correction

^b^Group main effects in generalised estimating equations

^c^Time and group interaction effects in generalised estimating equations

**Table S3. Differences between the two groups in primary outcomes in the** **original sample**

| Outcomes | Timepoint | Hi_tACS+MIRT  (n=25) | sham-Hi_tACS+MIRT  (n=25) | Effect size  (95% CI) | *P_FDR_* ^a^ | *P*-group^b^ | *P*-group*time^c^ |
| --- | --- | --- | --- | --- | --- | --- | --- |
| PDQ_39 total score, median (IQR) | T0 | 16.66(9.61,25.96) | 20.51(18.58,29.48) | 0.30(-0.26,0.86) |  | <0.001^*^ | .072 |
|  | T2 | 14.74(7.05,20.51) | 19.87(14.74,29.80) | 0.41(0.17,0.97) | 0.041 |  |  |
|  | T3 | 14.74(6.73,20.83) | 19.87(10.89,27.88) | 0.96(0.34,1.57) | 0.041 |  |  |
|  | T4 | 14.10(7.37,21.79) | 26.28(17.62,33.33) | 0.91(0.29,1.51) | 0.007 |  |  |

IQR = interquartile range; PDQ-39 = the 39-item Parkinson’s Disease Questionnaire

^a^Statistical differences between groups comparing at the same time point

^b^Group main effects in generalised estimating equations

^c^Time and group interaction effects in generalised estimating equations

**Table S4. Differences between the two groups in secondary outcomes in the ITT sample**

| Outcomes | Timepoint | Hi_tACS+MIRT  (n=30) | sham-Hi_tACS+MIRT  (n=30) | Effect size  (95% CI) | *P_FDR_* ^a^ | *P*-group^b^ | *P*-group*time^c^ |
| --- | --- | --- | --- | --- | --- | --- | --- |
| UPDRS-III, median (IQR) | T0 | 24.00(18.00, 32.50) | 23.50(17.75, 29.50) | -0.05 (-0.55,0.46) |  | NA | NA |
|  | T1 | 20.00(14.75, 26.25) | 19.00 (15.00, 26.00) | -0.05 (-0.55,0.46) | 0.853 |  |  |
| MPAS, median (IQR) | T0 | 87.00(82.00, 90.00) | 88.00(81.50,91.25) | -0.09(-0.65,0.46) |  | NA | NA |
|  | T1 | 89.00(84.00, 92.00) | 88.00(80.75, 92.00) | -0.36(-0.92,0.21) | 0.267 |  |  |
| BBS, median (IQR) | T0 | 54.50(54.00, 56.00) | 56.00(54.00,56.00) | -0.20(-0.75,0.36) |  | NA | NA |
|  | T1 | 55.00(55.00, 56.00) | 54.00(56.00, 56.00) | -0.23(-0.78,0.34) | 0.732 |  |  |
| TUG, median (IQR) | T0 | 8.50(8.00, 9.00) | 8.00(7.75, 10.00) | 0.60(0.02,1.18) |  | NA | NA |
|  | T1 | 8.00(7.75, 9.00) | 8.00(8.00, 10.00) | -0.02 (-0.58,0.53) | 0.392 |  |  |
| HAMA, median (IQR) | T0 | 12.00(7.00,15.00) | 14.00(7.00,19.25) | 0.45(-0.12,1.01) |  | 0.008 | 0.062 |
|  | T2 | 6.50(1.00,11.00) | 11.00(4.75,16.50) | 0.67(0.07,1.24) | 0.009 |  |  |
|  | T3 | 5.50(0.75,12.00) | 6.98(1.50,16.25) | 0.20(-0.39,0.72) | 0.372 |  |  |
|  | T4 | 5.50(2.00,11.25) | 8.50(3.75,18.75) | 0.89(0.33,1.43) | 0.035 |  |  |
| HAMD, median (IQR) | T0 | 10.50(6.75,15.00) | 12.50(6.75,17.25) | 0.10(-0.41,0.60) |  | 0.002 | 0.216 |
|  | T2 | 7.00(2.00,12.00) | 11.00(7.00,19.00) | 0.55(0.02,1.07) | 0.018 |  |  |
|  | T3 | 5.00(2.00,10.00) | 10.00(5.00,15.78) | 1.06(0.48,1.62) | 0.008 |  |  |
|  | T4 | 5.50(3.00,13.25) | 14.00(6.00,21.00) | 0.78(0.23,1.32) | 0.006 |  |  |
| GDS, median (IQR) | T0 | 9.00(2.75,18.00) | 9.00(5.00,15.25) | 0.08(-0.42,0.59) |  | 0.051 | 0.004^*^ |
|  | T2 | 8.00(3.75,12.00) | 9.50(2.00,15.00) | 0.24(-0.27,0.75) | 0.432 |  |  |
|  | T3 | 6.00(1.00,11.25) | 12.00(6.35,15.25) | 1.03(0.45,1.59) | 0.008^*^ |  |  |
|  | T4 | 5.00(2.00,17.00) | 11.50(4.75,19.25) | 0.59(0.06,1.11) | 0.050^*^ |  |  |
| NMSS total score, median (IQR) | T0 | 27.50(17.75,40.00) | 36.50(28.00,44.25) | 0.39(-0.13,0.90) |  | <0.001^*^ | 0.008^*^ |
|  | T2 | 10.50(0.00,22.25) | 35.50(21.25,51.00) | 0.92(0.36,1.48) | 0.016^*^ |  |  |
|  | T3 | 14.00(3.75,26.25) | 36.00(16.50,61.00) | 1.39(0.77,2.00) | 0.004^*^ |  |  |
|  | T4 | 14.50(3.00,29.00) | 46.50(26.75,55.25) | 1.37(0.75,1.98) | <0.001^*^ |  |  |
| MAES, median (IQR) | T0 | 13.00(8.75,17.25) | 14.50(9.00,18.25) | 0.09(-0.42,0.60) |  | 0.009^*^ | 0.028^*^ |
|  | T2 | 9.00(6.75,14.00) | 12.00(8.00,17.25) | 0.39(-0.13,0.90) | 0.086 |  |  |
|  | T3 | 9.50(4.00,16.00) | 13.50(9.23,21.25) | 0.68(0.14,1.22) | 0.045^*^ |  |  |
|  | T4 | 9.00(4.00,16.25) | 15.00(9.75,20.75) | 1.16(0.56,1.73) | 0.040^*^ |  |  |

IQR = interquartile range; UPDRS III = part 3 of the Movement Disorders-Unified Parkinson’s Disease Rating Scale; MPAS = Modified Parkinson Activity Scale; BBS = Berg Balance Scale; TUG = Timed Up and Go Test; HAMA = Hamilton Anxiety Scale; HAMD = Hamilton Depression Scale; GDS = Geriatric Depression Scale; NMSS = Non-Motor Symptoms Scale; MAES = Modified Apathy Estimate Scale

^a^Statistical differences between groups comparing at the same time point with FDR correction

^b^Group main effects in generalised estimating equations

^c^Time and group interaction effects in generalised estimating equations

**Table S5. Differences between the two groups in secondary outcomes in the original sample**

| Outcomes | Timepoint | Hi_tACS+MIRT  (n=25) | sham-Hi_tACS+MIRT  (n=25) | Effect size  (95% CI) | *P_FDR_* ^a^ | *P*-group^b^ | *P*-group*time^c^ |
| --- | --- | --- | --- | --- | --- | --- | --- |
| UPDRS-III, median (IQR) | T0 | 24.00(20.00,33.00) | 23.00(17.50,27.50) | -0.05(-0.61,0.50) |  | NA | NA |
|  | T1 | 20.00(14.75, 26.25) | 19.00 (15.00, 26.00) | -0.10(-0.65,0.46) | 0.853 |  |  |
| MPAS, median (IQR) | T0 | 87.00(82.00,90.00) | 88.00(81.00,91.50) | -0.09(-0.65, 0.46) |  | NA | NA |
|  | T1 | 88.00(84.00, 92.00) | 88.00(80.50, 92.00) | -0.36(-0.92,0.21) | 0.491 |  |  |
| BBS, median (IQR) | T0 | 55.00(54.00, 56.00) | 56.00(54.00,56.00) | -0.20(-0.75,0.36) |  | NA | NA |
|  | T1 | 56.00(55.00, 56.00) | 56.00(54.00, 56.00) | -0.23(-0.78,0.34) | 0.846 |  |  |
| TUG, median (IQR) | T0 | 8.00(8.00, 9.00) | 8.00(7.50, 10.00) | 0.60(0.02,1.18) |  | NA | NA |
|  | T1 | 8.00(7.00, 9.00) | 8.00(8.00, 10.00) | -0.02(-0.58,0.53) | 0.416 |  |  |
| HAMA, median (IQR) | T0 | 12.00(7.00,15.00) | 15.00(7.50,19.50) | 0.45(-0.12,1.01) |  | 0.008^*^ | 0.084 |
|  | T2 | 5.00(1.00,11.00) | 11.00(6.00,17.00) | 0.67(0.07,1.24) | 0.007 |  |  |
|  | T3 | 5.00(0.00,12.00) | 7.00(1.00,16.50) | 0.17(-0.39,0.72) | 0.359 |  |  |
|  | T4 | 6.00(3.00,12.00) | 8.00(3.50,20.50) | 0.55(-0.03,1.12) | 0.080 |  |  |
| HAMD, median (IQR) | T0 | 9.00(6.00,14.00) | 13.00(8.00,17.50) | 0.33(-0.23,0.89) |  | 0.001^*^ | 0.453 |
|  | T2 | 7.00(2.00,12.00) | 11.00(7.00,19.00) | 0.56(0.02,1.13) | 0.007 |  |  |
|  | T3 | 4.00(2.00,10.00) | 11.00(5.50,15.86) | 0.79(0.19,1.38) | 0.007 |  |  |
|  | T4 | 5.00(3.00,14.00) | 14.00(6.00,21.00) | 0.76(0.16,1.35) | 0.014 |  |  |
| GDS, median (IQR) | T0 | 8.00(2.00,18.00) | 9.00(5.00,15.50) | -0.21(-0.77,0.35) |  | 0.084 | 0.019^*^ |
|  | T2 | 6.00(3.00,12.00) | 10.00(2.00,16.00) | 0.02(-0.53,0.58) | 0.475 |  |  |
|  | T3 | 6.00(1.00,12.00) | 12.00(5.89,15.50) | 0.59(0.004,1.16) | 0.021^*^ |  |  |
|  | T4 | 9.00(2.00,17.00) | 12.00(4.50,20.50) | 0.40(-0.17,0.96) | 0.198 |  |  |
| NMSS total score, median (IQR) | T0 | 23.00(17.00,33.00) | 37.00(28.00,45.00) | 0.53(-0.05,1.10) |  | <0.001^*^ | 0.083 |
|  | T2 | 6.00(.00,22.00) | 23.00(7.50,39.00) | 0.78(0.17,1.37) | 0.016 |  |  |
|  | T3 | 15.00(4.00,27.00) | 36.00(16.00,57.00) | 1.06(0.43,1.69) | 0.009 |  |  |
|  | T4 | 15.00(3.00,32.00) | 41.00(27.00,56.00) | 1.15(0.50,1.78) | <0.001 |  |  |
| MAES, median (IQR) | T0 | 13.00(8.00,18.00) | 15.00(9.00,18.5) | -0.02(-0.58,0.53) |  | 0.023^*^ | 0.105 |
|  | T2 | 10.00(6.00,14.00) | 12.00(8.00,17.50) | 0.31(-0.26,0.87) | 0.165 |  |  |
|  | T3 | 11.00(4.00,16.00) | 13.00(9.15,21.50) | 0.56(0.02,1.13) | 0.050^*^ |  |  |
|  | T4 | 10.00(4.00,17.00) | 15.00(9.50,21.50) | 0.40(-0.17,0.97) | 0.080 |  |  |

IQR = interquartile range; UPDRS III = part 3 of the Movement Disorders-Unified Parkinson’s Disease Rating Scale; MPAS = Modified Parkinson Activity Scale; BBS = Berg Balance Scale; TUG = Timed Up and Go Test; HAMA = Hamilton Anxiety Scale; HAMD = Hamilton Depression Scale; GDS = Geriatric Depression Scale; NMSS = Non-Motor Symptoms Scale; MAES = Modified Apathy Estimate Scale

^a^Statistical differences between groups comparing at the same time point with FDR correction

^b^Group main effects in generalised estimating equations

^c^Time and group interaction effects in generalised estimating equations

| Participant ID | Group | Type of adverse event | Duration |
| --- | --- | --- | --- |
| 6 | Hi-tACS+MIRT | Itches | 2 days |
| 35 | sham-Hi-tACS+MIRT | Itches | 1 day |

**Table S6. Summary of the most common non-serious adverse events and duration experienced by participants in both groups**
